# Suspected Clinical Ellis-Yale-Winter Syndrome in an Infant With Unilateral Renal Agenesis, Contralateral Renal Ectopia, Microcephaly, and Congenital Heart Defects: A Case Report

**DOI:** 10.7759/cureus.119314

**Published:** 2026-11-08

**Authors:** Mara C Tejeda, Claudia A Garcia Gonzalez, Marisleydis Perez Santiago, Arturo D Somano Chaviano, Jorge A Nunez Garcia

**Affiliations:** 1 Hematology, Arnaldo Milian Clinical University Hospital, Villa Clara, CUB; 2 Critical Care, Faustino Perez Hospital, Matanzas, CUB; 3 Clinical Research, Primary Care - Research Initiative (PCRI), Miami, USA; 4 Pediatric Surgery, Jose Luis Miranda Pediatric University Hospital, Villa Clara, CUB

**Keywords:** congenital heart defects, ellis-yale-winter syndrome, microcephaly, multiple congenital anomalies, renal agenesis, renal ectopia

## Abstract

Ellis-Yale-Winter syndrome (EYWS) is an exceptionally rare multiple congenital anomaly syndrome characterized by microcephaly and congenital heart disease, variably associated with unilateral renal agenesis and abnormal pulmonary lobation. Its phenotype derives from a single original sibship, no validated diagnostic criteria exist, and no causative gene has been established.

We report a male infant with congenital microcephaly, right renal agenesis with a contralateral ectopic solitary kidney, congenital heart defects, craniofacial dysmorphic features, and neurological abnormalities. The findings showed phenotypic overlap with the original cases, leading a multidisciplinary team to a suspected clinical diagnosis of EYWS. This diagnosis is not molecularly confirmed, as no genetic testing was performed. Pulmonary lobation and segmentation could not be assessed, which is a major limitation; their presence or absence cannot be inferred. The contralateral renal ectopia may represent a coincidental malformation or a hypothetical phenotypic variant. Long-term outcome and vital status are undetermined.

This report does not establish new diagnostic features or an expanded phenotypic spectrum. Dedicated pulmonary imaging, exome or genome sequencing, DNA banking, and longitudinal follow-up are recommended in future cases.

## Introduction

Ellis-Yale-Winter syndrome (EYWS), also known as microcephaly-cardiac defect-lung malsegmentation syndrome, is an exceptionally rare congenital anomaly syndrome first described in 1996 in three sisters with microcephaly and congenital heart disease. Unilateral renal agenesis was reported in two siblings and abnormal pulmonary lobation in two of the three. The authors proposed an autosomal recessive inheritance pattern [1].

EYWS has remained poorly characterized in the decades since its initial description. The condition is cataloged in Online Mendelian Inheritance in Man (OMIM #601355) and Orphanet (ORPHA:2516), with an estimated prevalence of less than 1 per 1,000,000 [2,3]. No causative gene has been established [2,4]. A recent review, published nearly three decades after the original report, continues to base the clinical characterization of EYWS on the original three affected siblings [4]. Furthermore, our literature search identified no subsequent detailed clinical reports of affected individuals outside the originally described family. No validated diagnostic criteria or formal diagnostic checklist exist for EYWS. Consequently, the phenotypic spectrum and natural history of EYWS remain poorly defined, and its molecular basis remains unresolved.

We report a male infant with congenital microcephaly, heart defects, dysmorphic features, neurological abnormalities, right renal agenesis, and contralateral renal ectopia. Based on the overall phenotype, a multidisciplinary team made a suspected clinical diagnosis of EYWS, which relies on phenotypic overlap with the original sibship and on multidisciplinary clinical reasoning and is not molecularly confirmed. To our knowledge, this is the first detailed report outside the originally described family and the first reported affected male. The renal findings, particularly the contralateral ectopic kidney, may represent a coincidental malformation or a hypothetical phenotypic variant; they are not an established feature of EYWS and are presented as hypothesis-generating only. In the absence of molecular genetic testing and dedicated pulmonary imaging to assess lung lobation and segmentation, the diagnosis remains suspected, clinical, and phenotypic. This case contributes additional clinical observations to the limited literature on EYWS and highlights the need for further characterization of its phenotypic spectrum.

## Case presentation

Initial presentation

A male infant was born to a 33-year-old mother in a nonconsanguineous union. The pregnancy was considered high risk because of maternal hypertension during the third trimester and rapid maternal weight gain; there was no history of maternal diabetes. Prenatal ultrasonography performed at 31 weeks of gestation identified right renal agenesis and ectopia of the left kidney. Delivery occurred vaginally at 40 weeks and five days of gestation. At birth, the infant weighed 3,850 g, measured 50 cm in length, and had a head circumference of 31 cm (below the 3rd percentile). Apgar scores were 8 and 9 at one and five minutes, respectively.

At one month and 10 days of age, the patient was admitted after laboratory testing showed a plasma creatinine level of 58 μmol/L (0.66 mg/dL). Given the right renal agenesis and ectopic left kidney, he was referred to pediatric nephrology and diagnosed with chronic kidney disease (CKD).

Physical examination

Craniofacial examination showed a flat forehead, round low-set ears, preauricular pits, a short neck with skin folds, a high-arched palate, and protrusion of the tongue. No digital or spinal abnormalities were documented. Neurological examination revealed microcephaly with a head circumference of 33.3 cm (below the 3rd percentile), involuntary movements of the upper extremities, limb contractures, and cervical hyperextension. The heart rate was 120 beats/min and blood pressure was 130/90 mmHg. A grade V/VI systolic murmur was audible at the lower left sternal border without radiation.

Imaging studies

Renal Findings

Renal ultrasonography did not visualize the right kidney. The left kidney was ectopic, located in a low lumbar position near the midline, and measured 55 × 30 mm, with a parenchymal thickness of 12 mm. The urinary bladder was normal, with no ureteral dilatation or nephrolithiasis.

Cardiovascular Findings

Doppler echocardiography demonstrated balanced cardiac chambers, a small muscular ventricular septal defect, and a secundum atrial septal defect of approximately 2-3 mm with left-to-right shunting. A ductus arteriosus was not visualized, and the pulmonary trunk and its branches were not dilated (Figure 1).

**Figure 1:**
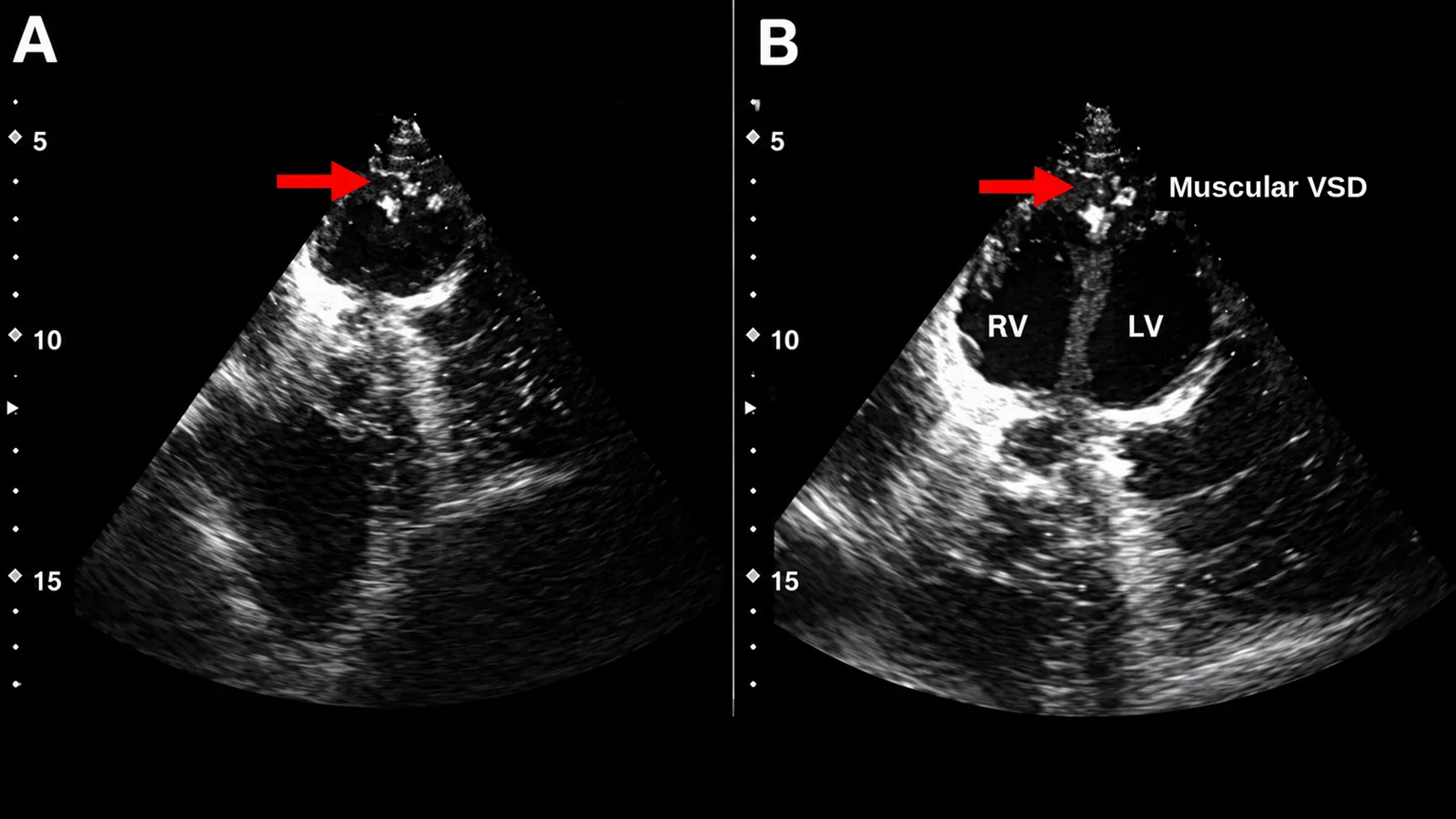
Transthoracic echocardiography, apical view (A, B) Small muscular ventricular septal defect (arrows). LV: leA ventricle; RV: right ventricle; VSD: ventricular septal defect.

Neurological Findings

Cranial computed tomography (CT) demonstrated marked ventricular dilatation, predominantly involving the lateral ventricles (right, approximately 23 mm; left, approximately 24 mm), with a rounded third ventricle of approximately 15 mm and a normal fourth ventricle. The supratentorial and infratentorial subarachnoid spaces were increased, particularly at the Sylvian fissures, the cisterna magna, and the periencephalic cistern. Hyperdensity of the thalami and midbrain was also noted and is considered a finding of uncertain significance (Figure 2).

**Figure 2:**
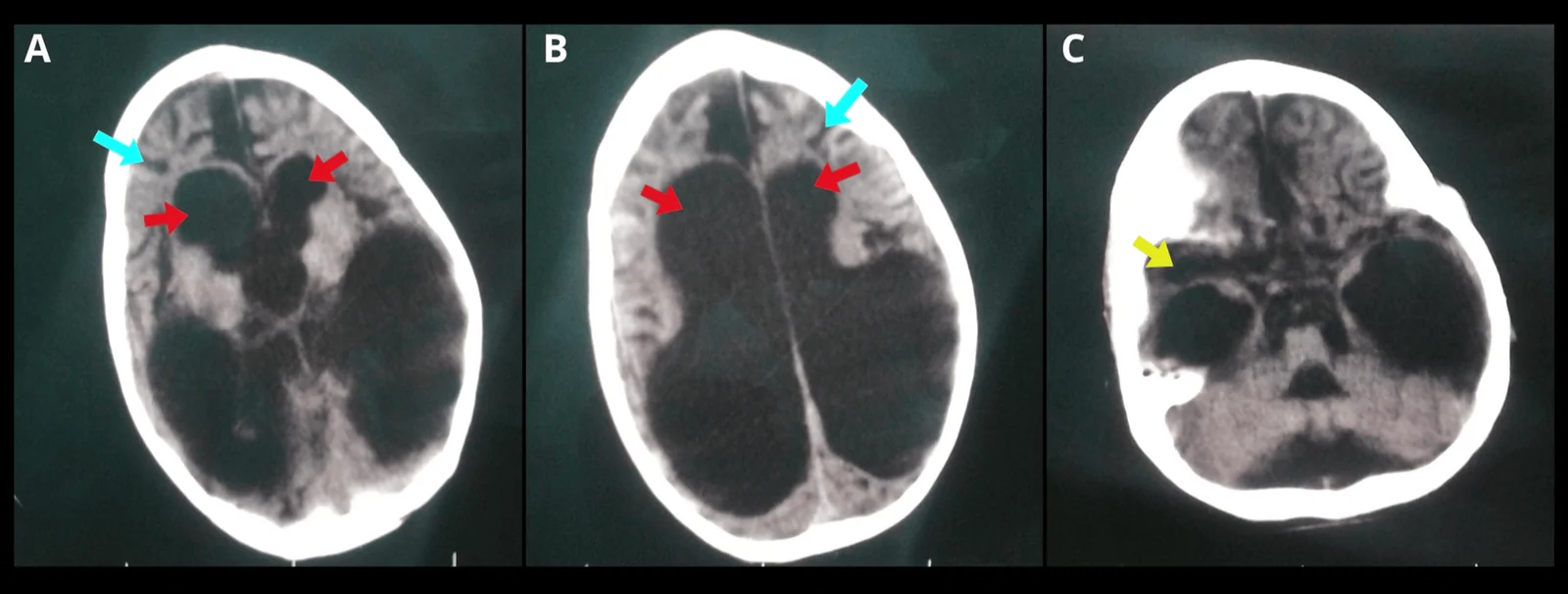
Non-contrast axial computed tomography (CT) of the brain (A, B) Marked bilateral lateral ventriculomegaly (red arrows), with prominence of the frontal subarachnoid spaces (cyan arrows). (C) Prominent subarachnoid/cisternal cerebrospinal fluid space (yellow arrow).

Clinical course and outcome

During hospitalization in the nephrology ward, the patient experienced a sudden aspiration episode while breastfeeding, followed by cardiopulmonary arrest. Cardiopulmonary resuscitation, endotracheal intubation, and mechanical ventilation were performed, and the patient was transferred to the pediatric intensive care unit. Subsequent cardiac assessment documented impaired myocardial contractility, suggestive of myocardial dysfunction, and a small posterior pericardial effusion of approximately 3 mm. The earlier respiratory history was limited to an infectious respiratory episode. Approximately two weeks later, the patient was successfully extubated, with good ventilatory mechanics, and was transferred to the intermediate care unit for continued monitoring and management. The last available clinical documentation corresponded to four months of age. No subsequent follow-up records were available, and the patient's long-term clinical outcome and current vital status could not be determined. The patient was not available for further clinical evaluation.

Based on the overall pattern of congenital anomalies and associated dysmorphic features, a multidisciplinary team made a suspected clinical diagnosis of EYWS. No chest imaging or radiology reports were available to retrospectively assess pulmonary lobation or segmentation. No genetic or molecular testing was performed. This suspected diagnosis was made clinically based on the phenotypic constellation observed in this patient and its correspondence with the limited clinical features previously reported for this rare condition. The principal findings supporting this suspected diagnosis included congenital microcephaly, structural congenital heart defects, and congenital renal abnormalities. However, given the extremely limited number of previously documented cases and the absence of validated diagnostic criteria or an established molecular marker, the diagnosis remains based on clinical and phenotypic assessment and is not molecularly confirmed. No formal diagnostic checklist exists; the diagnosis therefore relies on phenotypic overlap and multidisciplinary clinical reasoning.

## Discussion

EYWS was originally described in 1996 in three affected sisters with overlapping but variable combinations of microcephaly, congenital heart disease, pulmonary segmentation defects, renal abnormalities, and craniofacial anomalies [1]. A targeted review of the literature identified no additional detailed clinical case reports beyond the original sibship, and the 2025 review by Kazemzadeh and Razi continues to define the syndrome largely on the basis of those three patients [4]. Because the phenotype therefore derives from a single family and no causative gene has been established, its clinical boundaries remain poorly defined, and recognition of additional patients currently relies largely on phenotypic overlap with the original description [2,4]. No validated diagnostic criteria or formal diagnostic checklist exist for EYWS; the diagnosis in the present patient therefore relies on phenotypic overlap with the original sibship and on multidisciplinary clinical reasoning, and it must be regarded as a suspected clinical diagnosis that is not molecularly confirmed. Our male patient shares several major features, particularly congenital microcephaly, congenital heart disease, and unilateral renal agenesis. He additionally had ectopia of the contralateral solitary kidney, a renal configuration not reported in the original family [1]. The phenotypic comparison is summarized in Table 1, which uses the three siblings reported by Ellis et al. [1], the only primary source, later summarized in [4] as the benchmark, and classifies each feature in the present patient as concordant, partially concordant, additional, not documented, or unassessable. 

**Table 1:** Comparison of Clinical and Phenotypic Features Between the Original Ellis-Yale-Winter Syndrome Cases and the Present Case The benchmark comprises the three affected siblings described by Ellis et al. [1]. Features were classified as concordant if the same feature was present in both the benchmark and the present case; partially concordant if the features were related but not identical; additional if the feature was not reported in the benchmark; not documented if a feature reported in the benchmark was not recorded in the present case; and unassessable if the feature could not be evaluated or compared. ASD: atrial septal defect; VSD: ventricular septal defect.

Feature	Sibling 1	Sibling 2	Sibling 3 (fetus)	Present case	Classification	Interpretation
Sex	Female	Female	Female	Male	Additional	First reported male according to the available literature review; a descriptive difference only, with no inference on inheritance.
Microcephaly	Present	Present	Present	Present	Concordant	Present in all original siblings and in the present patient.
Congenital heart disease	Truncus arteriosus; membranous VSD	VSD	VSD	Small muscular VSD; secundum ASD (2–3 mm)	Partially concordant	VSD is shared; the VSD type differs and the ASD is an additional lesion.
Renal abnormalities	Right renal agenesis	Right renal agenesis	Both kidneys present	Right renal agenesis; ectopic left solitary kidney	Concordant (agenesis); additional (ectopia)	Agenesis is concordant with two original cases. Contralateral ectopia was not reported in the original cases; it may be a coincidental malformation or a hypothetical variant and is hypothesis-generating only.
Pulmonary lobation/segmentation	Abnormal lobation: right lung with two lobes; left lung unilobed	Not demonstrated	Right lung hypolobulation	Not assessed; no dedicated imaging or anatomical evaluation available	Unassessable	A core EYWS feature that could not be evaluated; presence or absence cannot be inferred.
Palatal abnormality	Posterior cleft palate	High-arched intact palate	Not reported	High-arched palate	Partially concordant	Similar to sibling 2 only; the specific palatal findings differ among the original siblings.
Ear/preauricular findings	Bilateral preauricular pits	Simple ears	Low-set ears	Round, low-set ears; preauricular pits	Partially concordant	Preauricular pits (sibling 1) and low-set ears (sibling 3) were each described in one original sibling.
Neck abnormalities	Short neck with redundant skin folds	Not reported	Neck webbing	Short neck with skin folds	Concordant (with sibling 1)	Cervical findings concordant with sibling 1.
Limb/digital abnormalities	Not reported	Not reported	Flexion deformities; short terminal phalanges	Limb contractures; no digital defects documented	Partially concordant	Contractures partially overlap with the flexion deformities of sibling 3; no digital abnormalities were documented.
Central nervous system abnormalities	Heterotopic nodules and malformed cortical areas	Hydranencephaly	Not reported	Marked dilatation of the lateral and third ventricles; enlarged subarachnoid spaces; thalamic and midbrain hyperdensity (uncertain significance)	Additional	Ventriculomegaly is not equivalent to hydranencephaly; the CT hyperdensities are of uncertain significance and have not been described in the original cases.
Other dysmorphic features	Micrognathia	Hypoplastic alae nasi; widely spaced/hypoplastic nipples	Not reported	Flat forehead; protruding tongue	Additional; original features not documented	Micrognathia, hypoplastic alae nasi, and cleft palate were not documented in the present patient.
Genetic/cytogenetic evaluation	Cytogenetic studies reported as normal in the original family	Same family investigation	Same family investigation	No molecular genetic testing performed	Unassessable	No causative molecular marker has been established; the lack of testing precludes any genetic comparison.
Outcome	Died at 16 days	Died at 13 months	Pregnancy terminated at 20 weeks	No follow-up records available; long-term outcome and vital status undetermined	Unassessable	Outcome cannot be compared.

Renal involvement is particularly relevant in the present case. Unilateral renal agenesis was documented in the two liveborn siblings in the original report, and our patient similarly had right renal agenesis, representing a direct point of concordance with the original EYWS phenotype [1,4]. However, the renal phenotype in our patient was more complex because of the contralateral solitary ectopic kidney, a configuration not described in the original family [1]. Given the very small number of reported patients and the absence of molecular confirmation, renal ectopia cannot currently be considered an established feature of EYWS. It may represent a coincidental congenital malformation or, hypothetically, a phenotypic variant, and it is presented as hypothesis-generating only; no association with the syndrome is inferred. A congenital solitary functioning kidney with structural abnormalities carries an increased risk of subsequent kidney injury compared with an anatomically normal solitary functioning kidney; current pediatric recommendations therefore favor risk-adapted long-term renal surveillance [5,6].

Microcephaly is one of the central features of EYWS and was evident from birth, with the head circumference remaining below the third percentile at one month of age. Neurological findings included abnormal involuntary movements, limb contractures, and cervical hyperextension. In contrast, the original report described hydranencephaly in one of the affected siblings [1], and the more recent review also lists hydranencephaly among the reported neurological manifestations of EYWS [4]. Ventriculomegaly and hydranencephaly are distinct structural abnormalities and are not equated here: hydranencephaly is characterized by near-complete replacement of the cerebral hemispheres by cerebrospinal fluid, whereas in ventriculomegaly or hydrocephalus a cortical mantle, although thinned, is preserved [7,8]. Cranial CT in our patient demonstrated marked enlargement of the lateral and third ventricles and increased subarachnoid spaces. These findings were therefore described as ventriculomegaly and are not considered equivalent to hydranencephaly. 

Hyperdensity of the thalami and midbrain was an additional radiological finding of uncertain significance. The CT was performed before the aspiration event and cardiopulmonary arrest. The finding was therefore present before the acute decompensation and cannot be attributed to that event or to its sequelae; whether it is related to a congenital process or to the suspected syndrome cannot be determined from the available documentation, and no pathological mechanism is assigned. The reported hyperdensity of the thalami and midbrain has not been described in the original cases or in the subsequent review and therefore cannot currently be considered a characteristic feature of EYWS [1,4].

Congenital heart disease was a consistent feature of the original EYWS sibship and provides another important point of comparison. Ellis et al. reported congenital heart disease among all three siblings, while the 2025 review identifies ventricular septal defect, truncus arteriosus, and abnormal aortic morphology among the cardiovascular abnormalities associated with the syndrome [1,4]. Our patient's cardiac involvement represents a comparatively less complex structural phenotype. Thus, the presence of a ventricular septal defect is concordant with the previously reported cardiac spectrum. The atrial septal defect, however, represents an additional cardiac lesion not specifically emphasized in the original description and subsequent summary. The impaired myocardial contractility and small pericardial effusion documented after cardiopulmonary arrest should be considered separately from the congenital malformations because they were identified only after that acute event and should therefore not automatically be interpreted as primary manifestations of EYWS.

The craniofacial findings showed additional phenotypic overlap with the original EYWS sibship. Our patient had a flat forehead, short, round, low-set ears, preauricular pits, a short neck with skin folds, a high-arched palate, and tongue protrusion. Several of these findings were also documented in the original family, including preauricular pits, neck abnormalities, and a high-arched palate [1]. The 2025 review likewise summarizes preauricular pits, high or narrow palate, cleft palate, micrognathia, hypoplastic alae nasi, and webbed neck among the reported craniofacial features of EYWS [4]. Notably, a high-arched palate was documented in one of the original affected siblings, providing a direct point of phenotypic overlap with our patient [1]. Other findings reported in the original family, including cleft palate, hypoplastic alae nasi, and micrognathia, were not documented in the present case [1].

The musculoskeletal findings showed partial overlap with the original EYWS phenotype. In the original report, flexion deformities and short terminal phalanges were documented in the third affected sibling, whereas the 2025 review summarizes joint stiffness and other musculoskeletal abnormalities among the reported manifestations [1,4]. In our patient, limb contractures were documented, providing a potential point of phenotypic overlap with the flexion deformities described in the original family. However, no digital or spinal abnormalities were documented, and short terminal phalanges were not reported.

Pulmonary lobation or segmentation abnormalities were documented in two of the three original siblings described by Ellis et al., and the 2025 review likewise includes abnormal pulmonary lobation among the reported manifestations of EYWS [1,4]. However, no dedicated imaging or anatomical assessment of pulmonary lobation or segmentation was available for retrospective evaluation in the present patient. Although the patient experienced a respiratory infectious episode and subsequently required mechanical ventilation following an aspiration event, these clinical events cannot be used to establish the presence of a congenital pulmonary lobation or segmentation abnormality. Consequently, pulmonary involvement in this patient remains unassessed, and its presence or absence cannot be determined. The impossibility of assessing pulmonary lobation and segmentation, a core feature of EYWS, is the major limitation of this report; neither its presence nor its absence is inferred. In any future patient with suspected EYWS, dedicated pulmonary imaging (such as chest computed tomography) or anatomical assessment is recommended.

Molecular testing was not available for this patient, limiting the ability to establish a molecular diagnosis or investigate alternative genetic etiologies. Although the original authors proposed an autosomal recessive disorder based on the familial occurrence [1], no causative gene has been identified to date, and current disease resources do not provide an established molecular cause for EYWS [2,4]. The fact that our patient was male, whereas all three originally described affected siblings were female, should be considered a descriptive difference rather than evidence for or against a particular inheritance mechanism, given the extremely limited number of reported cases and the absence of molecular confirmation. The available evidence is insufficient to infer the mode of inheritance or the chromosomal location of any underlying locus; in particular, X-linked inheritance is not supported by the available data and is not proposed here. In accordance with American College of Medical Genetics and Genomics (ACMG) guidance, exome or genome sequencing would be appropriate as a first- or second-tier diagnostic investigation in a child with multiple congenital anomalies, potentially helping identify an alternative or overlapping genetic condition and further clarify the underlying etiology of the multisystem phenotype [9]. We therefore recommend comprehensive genetic testing, including exome or genome sequencing, as part of the evaluation of future similar patients. Future investigations in similarly affected individuals should also consider preserving biological samples through DNA banking, when feasible, to facilitate molecular studies as knowledge of the syndrome advances.

Several genetic syndromes and congenital anomaly conditions were considered in the differential diagnosis based on the combination of renal, cardiac, craniofacial, neurological, and musculoskeletal findings. Table 2 summarizes, for each condition, the features present, the features absent or not assessed, an explicit clinical weighting, and the reasons why definitive exclusion was not possible. Branchio-oto-renal (BOR) spectrum disorder was an important consideration because it may involve preauricular pits, branchial anomalies, hearing impairment, and congenital renal malformations, including renal agenesis [10]. The coexistence of preauricular pits and renal abnormalities in our patient therefore raised this possibility. Two major clinical criteria (preauricular pits and renal anomalies) were documented [10,11]. However, no branchial cleft anomalies were documented, a formal audiological assessment was not available to evaluate hearing impairment, and microcephaly, structural congenital heart defects, and ventriculomegaly are not part of the core BOR spectrum phenotype. Whether the clinical criteria are fulfilled depends on how the round, low-set ears are interpreted and on unassessed items; BOR spectrum disorder was therefore weighted as the most relevant alternative diagnosis and could not be definitively excluded based on the available clinical information. Ellis-van Creveld (EVC) syndrome was also considered because congenital heart defects and oral abnormalities may occur in affected individuals [12]. Nevertheless, characteristic features such as postaxial polydactyly, disproportionate limb shortening, and nail or other ectodermal abnormalities were not documented in this patient, making the available phenotype less supportive of this diagnosis. EVC syndrome was weighted as less likely; however, because skeletal imaging, nail and dental examinations, and genetic testing were not available, and because no consensus clinical diagnostic criteria exist for EVC syndrome [12], it could not be definitively excluded. 

**Table 2:** Differential diagnosis with clinical weighting and reasons why definitive exclusion was not possible Weighting reflects the documented phenotype only and is not a probability estimate. ASD: atrial septal defect; CNS: central nervous system; EVC: Ellis-van Creveld; FISH: fluorescence in situ hybridization; MLPA: multiplex ligation-dependent probe amplification; VSD: ventricular septal defect.

Condition	Characteristic features	Features present in this patient	Features absent, not documented, or unassessed	Clinical weighting	Barriers to definitive exclusion
BOR spectrum disorder	Major criteria: second branchial arch anomalies, hearing loss, preauricular pits, auricular malformation, renal anomalies. Minor criteria: external, middle, or inner ear anomalies, preauricular tags, facial asymmetry, palatal abnormalities	Preauricular pits; renal anomalies (unilateral agenesis); round, low-set ears; high-arched palate	Branchial cleft anomalies not documented; hearing not assessed; no temporal bone imaging; family history not documented; microcephaly, structural heart defects, and ventriculomegaly are not core features	Moderate: two major criteria documented; a third major criterion or two minor criteria not documented	No audiological or temporal bone assessment, no family evaluation, no EYA1/SIX1/SIX5 testing
EVC syndrome	Postaxial polydactyly, limb shortening, nail dystrophy, dental anomalies, thoracic narrowing, congenital heart disease (atrial septal defect most frequent), upper lip defect; no consensus clinical criteria	Atrial and ventricular septal defects	Digital abnormalities explicitly absent; limb length, nails, dentition, and chest shape not documented; microcephaly and renal agenesis are not core features	Low	No skeletal imaging, no nail or dental examination, no gene testing
VACTERL association	Combination (usually at least three) of vertebral, anorectal, cardiac, tracheoesophageal, renal, and limb anomalies; a diagnosis of exclusion	Cardiac (VSD, ASD) and renal (agenesis, ectopia) anomalies: two components	No vertebral, anorectal, tracheoesophageal, or limb (radial ray) anomaly documented; spinal imaging unavailable; microcephaly and CNS findings are not component anomalies	Low: only two components documented	No spinal imaging; anorectal and tracheoesophageal evaluation not documented; genetic mimics not tested
Wolf-Hirschhorn syndrome	4p16.3 deletion; growth restriction, microcephaly, characteristic facies, seizures, hypotonia, developmental delay, septal heart defects, renal anomalies, cleft lip/palate	Microcephaly; septal heart defects; renal anomalies	Birth weight (3,850 g) and length (50 cm) within the expected range; characteristic facies, seizures, and hypotonia not documented	Low	No karyotype or chromosomal microarray; no electroencephalography or neurodevelopmental assessment
22q11.2 deletion syndrome	Conotruncal and septal heart defects, palatal anomalies, hypocalcemia/hypoparathyroidism, thymic hypoplasia, renal anomalies, facial dysmorphism	Septal heart defects; renal anomalies; high-arched palate; ear anomalies	No conotruncal defect documented; parathyroid and immune evaluation not documented	Low	No FISH, MLPA, or chromosomal microarray
Smith-Lemli-Opitz syndrome	DHCR7-related; microcephaly, 2-3 toe syndactyly, ptosis, cleft palate, heart defects, genital anomalies in males, renal anomalies, feeding difficulties, growth failure; elevated 7-dehydrocholesterol	Microcephaly; heart defects; renal anomalies; high-arched palate	No digital abnormalities documented; external genitalia and ptosis not documented	Low	No sterol analysis and no DHCR7 testing
Potter sequence	Oligohydramnios sequence due to severe renal dysfunction (typically bilateral): pulmonary hypoplasia, characteristic facies, limb positional deformities	Unilateral renal agenesis with a functioning ectopic contralateral kidney; limb contractures	No severe oligohydramnios or pulmonary hypoplasia documented; amniotic fluid volume not specifically recorded	Low	Pulmonary anatomy unassessed
Other chromosomal or monogenic multiple congenital anomaly syndromes	Variable	Multisystem anomalies	Not applicable	Cannot be weighted	No genetic testing of any kind (karyotype, microarray, exome/genome sequencing)

Other chromosomal and metabolic syndromes (Wolf-Hirschhorn syndrome, 22q11.2 deletion syndrome, and Smith-Lemli-Opitz syndrome) [13-15] were considered because they may present with overlapping congenital cardiac, craniofacial, neurological, or renal abnormalities; in each case, the characteristic associated features were either not documented or not supported by the documented phenotype (Table 2), and the available data did not favor any of them. VACTERL association [16] was considered because it combines vertebral, anorectal, cardiac, tracheoesophageal, renal, and limb anomalies and is generally considered when at least three of its six components are present; only two (cardiac and renal) were documented. Potter sequence [17] was considered in relation to the renal malformation; however, unilateral renal agenesis with a contralateral functioning ectopic kidney, in the absence of documented severe oligohydramnios or pulmonary hypoplasia, did not support its classic presentation. None of these alternatives could be definitively excluded without comprehensive clinical assessment, chromosomal analysis, metabolic testing, and molecular genetic testing.

Overall, the absence of several characteristic findings made these alternative diagnoses less strongly supported by the documented phenotype, although none could be definitively excluded without comprehensive clinical assessment and molecular genetic testing. Following multidisciplinary evaluation, the combination of congenital microcephaly, congenital heart defects, unilateral renal agenesis, and craniofacial and other dysmorphic features was considered most consistent with a suspected clinical diagnosis of Ellis-Yale-Winter syndrome (EYWS), based on its phenotypic similarity to the originally reported family. This interpretation should nevertheless be viewed in the context of the limited number of reported cases and the absence of established molecular diagnostic criteria.

The documented findings of this report are the clinical and imaging observations described above. Separately, it can be hypothesized that the co-occurrence of renal agenesis, renal ectopia, congenital heart defects, microcephaly, and ventriculomegaly reflects a shared disturbance of early embryonic development, or alternatively that some of these anomalies arose independently or coincidentally. The present data cannot distinguish between these possibilities; no unifying etiology, developmental field defect, or causal relationship is inferred.

The patient required intensive care following aspiration and cardiopulmonary arrest and subsequently showed documented myocardial dysfunction; these acute events cannot be attributed to the suspected syndrome or to the congenital anomalies on the basis of the available data. The available records end at approximately four months of age, and no subsequent follow-up information is available; the long-term outcome and vital status are therefore undetermined, and no prognostic conclusion is drawn for this patient. The outcomes of the previously reported siblings cannot be extrapolated to an individual patient given the very small number of documented cases [1,4]. Longitudinal multidisciplinary follow-up (cardiology, nephrology with risk-adapted renal surveillance, neurology and neurodevelopment, and pulmonology) is recommended for similar patients.

This case has several important limitations. First, no genetic or molecular testing was performed, precluding molecular confirmation of the suspected clinical diagnosis. Second, pulmonary anatomy could not be retrospectively evaluated, which is a major limitation because pulmonary lobation and segmentation abnormalities are a core feature of the syndrome. Third, long-term follow-up was unavailable. Fourth, the patient was unavailable for further clinical evaluation and no retrospective assumptions were made. Future clinical and molecular follow-up, together with DNA banking, is recommended where feasible. Fifth, the phenotypic benchmark derives from a single original sibship, and no validated diagnostic criteria exist. Finally, the extreme rarity of EYWS and the small number of reported patients make it difficult to distinguish core manifestations from variable or coincidental findings.

Despite these limitations, the present case demonstrates a multisystem pattern that overlaps with the phenotype originally described by Ellis et al. and subsequently summarized by Kazemzadeh and Razi [1,4]. In particular, the coexistence of microcephaly, congenital heart disease, and unilateral renal agenesis, together with additional craniofacial and neurological abnormalities, supports a suspected clinical diagnosis of Ellis-Yale-Winter syndrome, which is not molecularly confirmed. The contralateral renal ectopia and the distinct neurological findings represent additional observations whose relationship to EYWS remains uncertain. Accordingly, this case should be interpreted as a possible contribution to the phenotypic characterization of the syndrome rather than definitive evidence of an expanded clinical spectrum. Further molecular characterization and longitudinal clinical evaluation of similarly affected patients will be essential to clarify the genetic basis, phenotypic boundaries, inheritance pattern, and prognosis of this exceptionally rare multiple congenital anomaly syndrome.

## Conclusions

This case describes a male infant with a multisystem congenital anomaly phenotype showing substantial overlap with the features originally described in EYWS, including congenital microcephaly, congenital heart disease, unilateral renal agenesis, craniofacial abnormalities, and neurological involvement. These findings support the clinical consideration of EYWS based on phenotypic similarity with the originally reported family, although the diagnosis remains suspected and clinical and is not molecularly confirmed. The contralateral ectopic solitary kidney and the distinct neurological findings, particularly ventriculomegaly rather than the hydranencephaly described in the original cases, represent additional observations whose relationship to EYWS remains uncertain. They may reflect phenotypic variability or coincidental congenital abnormalities and require future validation; they are not established features of the syndrome. The absence of molecular genetic confirmation and dedicated pulmonary imaging further limits definitive phenotypic characterization. Therefore, this report does not establish new diagnostic features or an expanded phenotypic spectrum. Additional cases, comprehensive genetic evaluation, including exome or genome sequencing and DNA banking, systematic assessment of pulmonary and renal abnormalities with dedicated pulmonary imaging, and longitudinal follow-up are needed to clarify the clinical spectrum of this exceptionally rare condition.
