# Correction: Suspected Clinical Ellis-Yale-Winter Syndrome in an Infant With Unilateral Renal Agenesis, Contralateral Renal Ectopia, Microcephaly, and Congenital Heart Defects: A Case Report

**DOI:** 10.7759/cureus.c502

**Published:** 2026-11-11

**Authors:** Mara C Tejeda, Claudia A Garcia Gonzalez, Marisleydis Perez Santiago, Arturo D Somano Chaviano, Jorge A Nunez Garcia

**Affiliations:** 1 Hematology, Arnaldo Milian Clinical University Hospital, Villa Clara, CUB; 2 Critical Care, Faustino Perez Hospital, Matanzas, CUB; 3 Clinical Research, Primary Care - Research Initiative (PCRI), Miami, USA; 4 Pediatric Surgery, Jose Luis Miranda Pediatric University Hospital, Villa Clara, CUB

Due to a technical error at the time of publication, the majority of the case presentation along with both figures were omitted from the final published version. This article has been corrected to restore the full case presentation with figures. 

